# Impact of Abdominal Aortic Calcification on Long-Term Outcome after Gastric Cancer Surgery: a Retrospective Study

**DOI:** 10.1007/s12029-025-01339-0

**Published:** 2025-11-15

**Authors:** Akihiro Kohata, Kazuaki Tanabe, Hidetoshi Shidahara, Shoko Kohata, Nozomi Karakuchi, Yuki Takemoto, Emi Chikuie, Hiroshi Ota, Yoshihiro Saeki, Hideki Ohdan

**Affiliations:** Department of Gastroenterological and Transplant Surgery, Graduate School of Biomedical and Health Sciences, Hiroshima University, Hiroshima, Japan

**Keywords:** Abdominal aortic calcification, Prognosis, Recurrence, Gastric cancer, Survival analysis

## Abstract

**Purpose:**

This study examined the impact of abdominal aortic calcification, a known risk factor for cardiovascular disease, on the prognosis of patients undergoing radical surgery for gastric cancer.

**Methods:**

The effects of abdominal aortic calcification on clinical outcomes, prognosis, and recurrence patterns were analyzed in 516 patients who underwent radical surgery for gastric cancer between 2010 and 2017.

**Results:**

After propensity score matching, patients with higher abdominal aortic calcification had significantly poorer overall survival (OS; *P* = 0.020), disease specific survival (DSS; *P* = 0.013), and recurrence-free survival (RFS; *P* = 0.017) than those with lower calcification levels. Multivariate Cox regression analysis identified a higher degree of abdominal aortic calcification as an independent risk factor for poor OS (hazard ratio, 2.57; 95% confidence interval, 1.56–4.22; *P* < 0.001), DSS (hazard ratio, 4.32; 95% confidence interval, 1.84–10.12; *P* < 0.001) and RFS (hazard ratio, 2.63; 95% confidence interval, 1.60–4.33; *P* < 0.001). High abdominal aortic calcification was also a risk factor for peritoneal dissemination recurrence in gastric cancer.

**Conclusion:**

A high degree of abdominal aortic calcification was linked to poor prognosis and might increase peritoneal dissemination recurrence following curative resection for gastric cancer. Thus, abdominal aortic calcification may serve as a novel clinical tool for predicting the prognosis of patients with gastric cancer.

**Supplementary Information:**

The online version contains supplementary material available at 10.1007/s12029-025-01339-0.

## Introduction

 Gastric cancer is the fifth most common malignant neoplasm worldwide and the third leading cause of cancer-related mortality [1, 2]. Although its incidence has risen with an aging population, the 5-year OS rate among older patients remains considerably lower than that of younger patients [3, 4]. This highlights the importance of evaluating surgical indications based on long-term prognosis along with preoperative assessments of nutritional status and frailty to identify high-risk cases [5–7].

Abdominal aortic calcification (AAC) is a known marker of atherosclerosis. Studies have linked AAC to increased risks of ischemic heart disease, stroke, and chronic inflammation, as reflected by markers such as the systemic immune-inflammation index and the pan-immune-inflammation value [8–10].

Additionally, AAC has been identified as a perioperative risk factor even in patients without other cardiovascular conditions [11]. In the upper gastrointestinal tract, AAC has been associated with short-term prognoses such as anastomotic leakage after esophageal surgery and gastrectomy [12, 13].

Emerging evidence suggests that AAC may impact long-term oncologic outcomes. While previous studies have explored the relationship between AAC and prognosis following colorectal cancer surgery [14], the potential link between AAC and long-term outcomes after gastric cancer surgery remains unexplored.

This study aims to investigate whether AAC scores are associated with long-term postoperative outcomes in patients with gastric cancer.

## Methods

### Patients

**Fig. 1:**
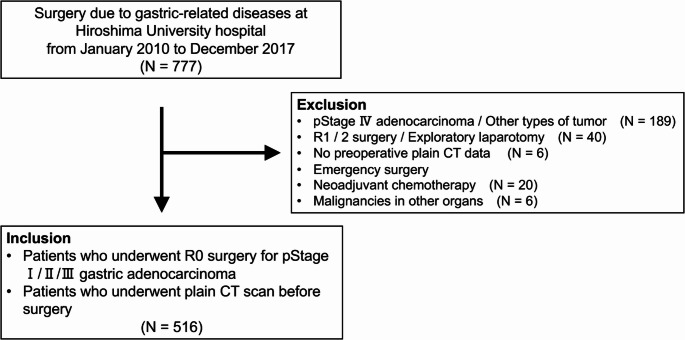
Inclusion and exclusion criteria for patient selection

Patient data of 777 individuals who underwent surgery for gastric-related diseases at the Department of Upper Gastrointestinal Surgery, Hiroshima University Hospital (January 2010 to December 2017), were collected from the hospital database. This study was approved by the Ethics Committee of Hiroshima University Hospital (approval number E2019-1789). The requirement for informed consent was waived due to the retrospective nature of the study. Patients were included if they underwent complete resection for pathological stage I/II/III (pStage I/II/III) gastric adenocarcinoma and plain computed tomography (CT) before surgery. Preoperative CT scans were performed at a median of 24 days prior to surgery (interquartile range: 15 to 33.75 days). Patients were excluded if they had a pathological diagnosis of stage IV, underwent incomplete resection or exploratory laparotomy, lacked preoperative plain CT imaging, underwent emergency surgery, had malignancies in other organs, or received neoadjuvant chemotherapy. The inclusion and exclusion criteria for patients with gastric cancer are shown in Fig. 1.

Surgery was performed using open, laparoscopic, and robotic approaches. Clinicodemographic data at the time of gastrectomy, including age, sex, body mass index (BMI), American Society of Anesthesiologists Physical Status (ASA-PS), Prognostic Nutritional Index (PNI), the C-reactive protein/albumin ratio (CAR), diabetes mellitus (DM), hypertension (HT), and hyperlipidemia (HL), were obtained from electronic records, along with pathological findings, including pStage, tumor location, tumor size and histological type, and surgical data, such as operation time and bleeding volume. Blood sample data collected within 30 days prior to surgery were used to calculate PNI and CAR. The tumor location was classified as Proximal (upper third of the stomach), Middle (middle third), or Distal (lower third) based on the position relative to the center of the primary tumor. Histological classification followed the Lauren classification. Data regarding recurrence and long-term postoperative survival were also extracted. After surgery, fluoropyrimidine-based adjuvant chemotherapy was primarily administered to patients with pStage III disease in accordance with Japanese guidelines for gastric cancer treatment. Adjuvant chemotherapy was also considered for patients with pStage II disease according to this guideline; however, not all eligible patients received chemotherapy as dosing was determined based on performance status and comorbidities. Follow-ups were conducted at intervals of 3–6 months for 5 years using contrast-enhanced CT and endoscopy.

### Aortic Abdominal Calcification

To quantify AAC, we adopted the Agatston score, a dimensionless index [15]. The score was measured using AZE VirtualPlace Lexus64 Anatomia software (AZE, Schaumburg, IL, USA), and the extent of calcification in the abdominal aorta was quantified on plain axial CT images at 5 mm intervals from the diaphragmatic leg to the level of the bifurcation of either the left or right renal artery. The Agatston score was consistently measured by one experienced surgeon. To ensure the validity of the measurement, the data of 30 randomly selected cases were re-evaluated by the same examiner at least one month after the initial measurement, and the intraclass correlation coefficient (ICC) was calculated, revealing a very high correlation (ICC = 0.882). Furthermore, to assess inter-rater reliability, the same 30 cases were independently evaluated by a second experienced surgeon, who was blinded to the first examiner’s results. This also demonstrated a high level of agreement (ICC = 0.982).

### Definition of Postoperative Complication

Postoperative complications were assessed according to the Clavien-Dindo classification [16] and included not only anastomotic leakage and pancreatic fistula but also other complications, such as pneumonia, intraperitoneal abscess/surgical site infection, and delayed gastric emptying (all grade II or higher).

### Statistical Analysis

The definitions for the survival endpoints were as follows. OS was calculated from the date of surgery to the date of death from any cause. Data for patients who were alive at the last follow-up were censored. DSS was defined from the date of surgery to the date of death due to gastric cancer. Data of patients who were alive at the last follow-up and patients who died of other causes were censored. RFS was defined as the time from the date of surgery to the first event of either tumor recurrence or death from any cause. Patients alive without recurrence at the last follow-up were censored. OS, DSS, and RFS were plotted using Kaplan–Meier analysis and compared using log-rank statistics. The duration of follow-up was 60 months or until death.

Multivariate analyses were conducted for variables independently associated with OS, DSS, and RFS using the Cox proportional hazards model. To assess whether AAC was an independent prognostic factor for patients after gastric cancer surgery, a multivariate analysis was performed using the Cox proportional hazards model. The model was adjusted for potential prognostic factors that have been established in previous literature. Based on the findings of prior large-scale cohort studies and meta-analyses, the following covariates were selected and included in the model: age, sex, pStage, histopathological type, tumor location, and operative method [17–20].

To adjust for differences in baseline characteristics, 1:1 propensity score models were constructed. Propensity scores were calculated using logistic regression including the following covariates: age, sex, BMI, ASA-PS, DM, HT, HL, PNI, CAR, pStage, tumor location, histopathological type, surgical method, lymph node dissection, and adjuvant chemotherapy. 1:1 nearest-neighbor matching without replacement was performed using a caliper width of 0.2 standard deviations of the logit of the propensity score.

All statistical analyses were performed using JMP version 18.0 (SAS Institute, Cary, NC, USA). Statistical significance was set at *p* < 0.05. Cutoff values for continuous variables such as CAR and Agatston score were established using receiver operating characteristic (ROC) curve analysis.

## Results

### Patient Characteristics

After applying the exclusion criteria, a total of 516 patients were included in this study. Using the ROC curve for 5-year survival rate as the outcome, the Agatston score cutoff value of 100 yielded an AUC of 0.6656 (95% CI: 0.5984–0.7267), with a sensitivity of 65.0% and specificity of 62.8%. Based on this, the patients were divided into two groups: a high AAC group with scores > 100 and a low AAC group with Agatston scores < 100. The high AAC group comprised 214 patients (41.5%), and the low AAC group included 302 patients (58.5%).

Compared with patients in the low AAC group, patients in the high AAC group were older and more likely to be men; frequently presented with DM, HT, or HL; had lower ASA-PS scores and PNI values but higher CAR; underwent open surgery more often; exhibited greater intraoperative blood loss; had more postoperative pneumonia; and more commonly exhibited the intestinal type according to the histological classification of the tumor. The clinical characteristics of both groups are summarized in Table 1.

**Table 1:** Patient characteristics in the low and high AAC groups

	N=516	Low AAC group (N=302)	High AAC group (N=214)	P value
Age	68 (21–89)	63 (21–87)	73 (50–89)	<0.001
Sex				0.001
Male	359 (69.6%)	192 (63.6%)	167 (78.0%)	
Female	157 (30.4%)	110 (36.4%)	47 (22.0%)	
BMI	22.1 (14.5-32.9)	22.0 (15.0-32.9)	22.3 (14.5-30.5)	0.864
ASA-PS				<0.001
1	50 (9.7%)	40 (13.2%)	10 (4.7%)	
2	434 (84.1%)	256 (84.8%)	178 (83.2%)	
3	32 (6.2%)	6 (2.0%)	26 (12.1%)	
Comorbidities				
DM	75 (14.5%)	28 (9.3%)	47 (22.0%)	<0.001
HT	155 (30.0%)	61 (20.2%)	94 (43.9%)	<0.001
HL	69 (13.4%)	31 (10.3%)	38 (17.8%)	0.018
Inflammation and nutritional markers				
PNI	50.5 (26.5–75.1)	51.1 (27.5–75.1)	49.3 (26.5–67.5)	0.001
NLR	1.99 (0.51–13.83)	1.96 (0.66–10.50)	2.01 (0.51–13.83)	0.512
CAR	0.02 (0.00–1.97)	0.01 (0.00–0.99)	0.02 (0.00–1.97)	0.001
Operative method				0.096
DG	333 (64.5%)	208 (68.9%)	125 (58.4%)	
PG	52 (10.1%)	25 (8.3%)	27 (12.6%)	
PPG	9 (1.7%)	5 (1.6%)	4 (1.9%)	
TG	122 (23.7%)	64 (21.2%)	58 (27.1%)	
Operative Approach				0.013
Laparoscopic, Robotic	318 (61.6%)	200 (66.2%)	118 (55.1%)	
Open	198 (38.4%)	102 (33.8%)	96 (44.9%)	
Dissection				0.096
D1	36 (7.0%)	15 (5.0%)	21 (9.8%)	
D1+	336 (65.1%)	199 (65.9%)	137 (64.0%)	
D2	144 (27.9%)	88 (29.1%)	56 (26.2%)	
Operating time (min)	299 (142–885)	298 (169–885)	300.5 (142–651)	0.388
Bleeding (mL)	55.5 (0–6882)	50 (0–1550)	82.5 (0–6882)	0.021
Postoperative complication				
Anastomotic leak	34 (6.6%)	15 (5.0%)	19 (8.9%)	0.104
Pancreatic fistula	18 (3.5%)	7 (2.3%)	11 (5.1%)	0.094
Abdominal abscess / surgical site infection	26 (5.0%)	16 (5.3%)	10 (4.7%)	0.84
Delayed gastric emptying	17 (3.3%)	11 (3.6%)	6 (2.8%)	0.803
Pneumonia	17 (3.3%)	4 (1.3%)	13 (6.1%)	0.005
pStage				0.245
Ⅰ	396 (76.7%)	232 (76.8%)	164 (76.6%)	
Ⅱ	54 (10.5%)	36 (11.9%)	18 (8.4%)	
Ⅲ	66 (12.8%)	34 (11.3%)	32 (15.0%)	
pT				0.052
T1	366 (70.9%)	218 (72.2%)	148 (69.2%)	
T2	60 (11.6%)	31 (10.3%)	29 (13.6%)	
T3	46 (8.9%)	33 (10.9%)	13 (6.1%)	
T4	44 (8.5%)	20 (6.6%)	24 (11.2%)	
pN				0.877
N0	397 (76.9%)	236 (78.2%)	161 (75.2%)	
N1	50 (9.7%)	27 (8.9%)	23 (10.8%)	
N2	34 (6.6%)	19 (6.3%)	15 (7.0%)	
N3	35 (6.8%)	20 (6.6%)	15 (7.0%)	
Tumor location				0.236
Proximal	136 (26.4%)	72 (23.8%)	64 (29.9%)	
Middle	181 (35.1%)	113 (37.4%)	68 (31.8%)	
Distal	199 (38.6%)	117 (38.7%)	82 (38.3%)	
Tumor size (mm)	25 (4-200)	25 (5-120)	25 (4-200)	0.573
Histopathological type				<0.001
Intestinal	240 (46.5%)	114 (37.8%)	126 (58.9%)	
Diffuse/mixed	276 (53.5%)	188 (62.3%)	88 (41.1%)	
Adjuvant chemotherapy	94 (18.2%)	57 (18.9%)	37 (17.3%)	0.729

*BMI* body mass index, *ASA-PS* American Society of Anesthesiologists Physical Status, *DM* diabetes mellitus, *HT* hypertension, *HL* hyperlipidemia, *PNI* Prognostic Nutritional Index, *NLR* Neutrophil-Lymphocyte Ratio, *CAR* C-reactive protein/Albumin Ratio, *DG* distal gastrectomy, *PG* proximal gastrectomy, *PPG* pylorus preserving gastrectomy, *TG* total gastrectomy, *AAC* abdominal aortic calcification

### Kaplan–Meier Survival Curve Analysis after Propensity Score Matching between the High and Low AAC Groups

Kaplan–Meier survival curve analysis was performed in a propensity score-matched cohort to evaluate the prognostic impact of AAC. Propensity score matching was applied to minimize potential confounding factors and achieve comparability between the high and low AAC groups in terms of baseline clinical and pathological characteristics that may influence long-term oncological outcomes. As a result, 143 patients were matched in each group, yielding a well-balanced cohort for subsequent survival analysis **(**Table 2**)**.

**Table 2:** Patient characteristics after propensity score matching

	Before propensity score matching			After propensity score matching		
	Low AAC group	High AAC group	P value	Low AAC group	High AAC group	P value
	N=302	N=214		N=143	N=143	
Age (>75)	35 (11.6%)	88 (41.1%)	<0.001	34 (23.8%)	34 (23.8%)	1
Sex (Male)	192 (63.6%)	167 (78.0%)	0.005	108 (75.5%)	109 (76.2%)	1
BMI (>25)	57 (18.9%)	41 (19.2%)	1	31 (21.7%)	26 (18.2%)	0.554
ASA-PS (>2)	6 (2.0%)	26 (12.2%)	<0.001	6 (4.2%)	6 (4.2%)	1
DM	28 (9.3%)	47 (22.0%)	<0.001	23 (16.8%)	23 (16.8%)	1
HT	61 (20.2%)	94 (43.9%)	<0.001	53 (37.1%)	52 (36.4%)	1
HL	31 (10.3%)	38 (17.8%)	0.018	19 (13.3%)	22 (15.4%)	0.736
PNI (<40)	17 (5.6%)	31 (14.6%)	0.001	11 (7.7%)	13 (9.1%)	0.832
CAR (>0.0205)	96 (31.8%)	109 (51.2%)	<0.001	62 (43.4%)	61 (42.7%)	1
pStage III	34 (11.3%)	32 (15.0%)	0.23	15 (10.5%)	19 (13.3%)	0.584
Tumor location (Proximal)	72 (23.8%)	64 (29.9%)	0.129	30 (21.0%)	37 (25.9%)	0.402
Histopathological type (Diffuse/mixed)	188 (62.3%)	88 (41.1%)	<0.001	69 (48.3%)	67 (46.9%)	0.906
Surgical method（TG）	64 (21.2%)	58 (27.1%)	0.141	33 (23.1%)	30 (21.0%)	0.776
Dissection (D2)	88 (29.1%)	56 (26.2%)	0.487	37 (25.9%)	42 (29.4%)	0.597
Adjuvant chemotherapy	57 (18.9%)	37 (17.3%)	0.729	21 (14.7%)	24 (16.8%)	0.746

*BMI* body mass index, *ASA-PS* American Society of Anesthesiologists Physical Status, *DM* diabetes mellitus, *HT* hypertension, *HL* hyperlipidemia, *PNI* Prognostic Nutritional Index, *CAR* C-reactive protein/Albumin Ratio, *TG* total gastrectomy, *AAC* abdominal aortic calcification

**Fig. 2:**
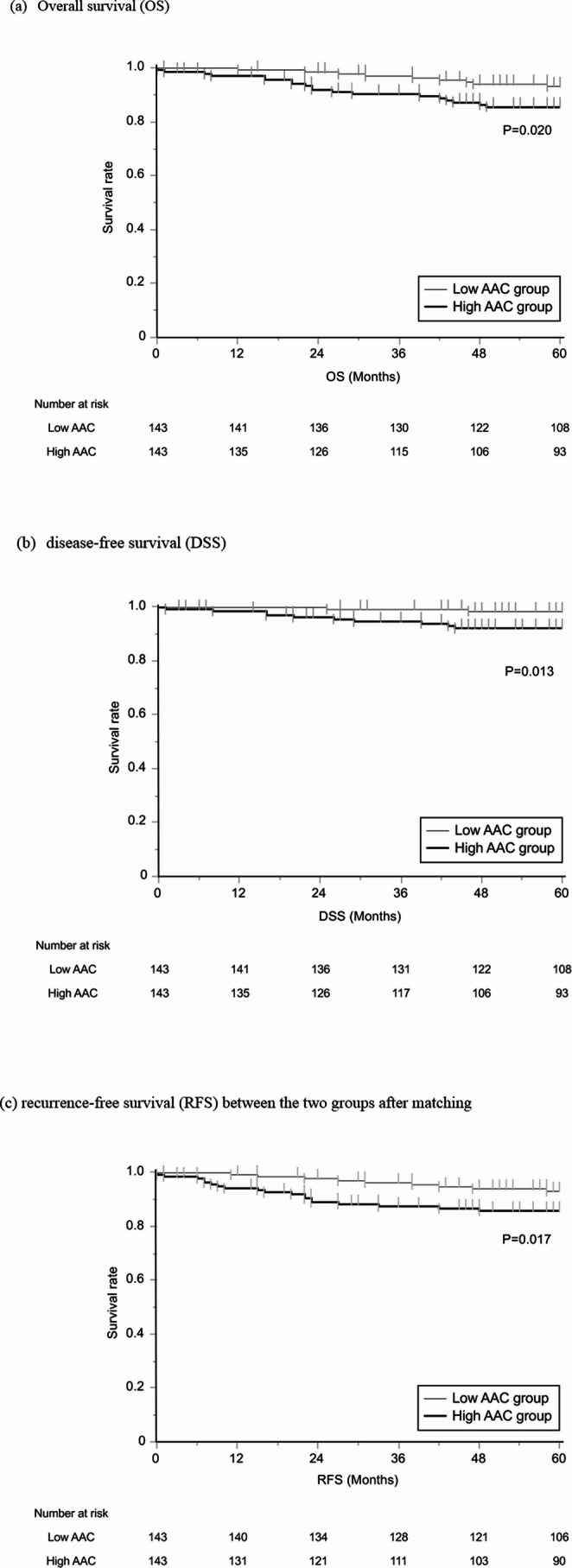
Kaplan-Meier survival curves. (**a**) Overall survival (OS), (**b**) disease-free survival (DSS), (**c**) recurrence-free survival (RFS) between the two groups after matching

In the matched population, Kaplan–Meier analysis demonstrated that patients in the high AAC group had significantly poorer OS (*P* = 0.020), DSS (*P* = 0.013), and RFS (*P* = 0.017) compared to those in the low AAC group (Fig. 2a–c). These findings suggest that the degree of AAC, as quantified by the Agatston score, may serve as an independent risk factor associated with adverse postoperative outcomes in patients undergoing curative resection for gastric cancer.

### Factors Related To OS, DSS, and RFS

The prognostic factors were analyzed using univariate analysis to evaluate patient background as well as oncological and surgical variables in relation to survival outcomes.

Univariate analysis identified high AAC, older age, male sex, high ASA-PS, low PNI, high CAR, pStage 3, proximal tumor, total gastrectomy, D2 dissection, and postoperative complications as predictors of the 5-year OS (Supplemental Table 1). Multivariate analysis confirmed older age (hazard ratio [HR], 2.01; 95% confidence interval [CI], 1.21–3.36; *P* = 0.007), pStage3 (HR, 2.62; 95% CI, 1.58–4.36; *P* < 0.001), total gastrectomy (HR, 2.65; 95% CI, 1.59–4.41; *P* < 0.001), and high AAC (HR, 2.57, 95% CI, 1.56–4.22, *P* < 0.001) as independent predictors of 5-year OS **(**Table 3**)**.

**Table 3:** Risk factors for 5-year OS

		Multivariate		
	N=516	HR	95% CI	P value
Age (>75)	123 (23.8%)	2.01	1.21-3.36	0.007
Sex (Male)	359 (69.6%)	1.83	0.99-3.40	0.055
pStage III	66 (12.8%)	2.62	1.58-4.36	<0.001
Histopathological type (Diffuse/mixed)	276 (53.5%)	1.14	0.72-1.80	0.567
Tumor location (Proximal)	136 (26.4%)	1.1	0.66-1.82	0.725
Surgical method （TG)	122 (23.6%)	2.65	1.59-4.41	<0.001
AAC (>100)	214 (41.5%)	2.57	1.56-4.22	<0.001

*TG* total gastrectomy, *AAC* abdominal aortic calcification, *OS* overall survival, *HR* hazard ratio, *CI* confidence interval

Univariate analysis identified the following predictors of poor DSS: high AAC, high ASA-PS, low PNI, high CAR, pStage 3, proximal tumor, total gastrectomy, D2 dissection, postoperative complications, and adjuvant chemotherapy (Supplemental Table 2). Multivariate analysis further identified the following factors as independent predictors of poor DSS: pStage 3 (HR, 6.26; 95% CI, 2.94–13.36; *P* < 0.001), total gastrectomy (HR, 5.38; 95% CI 2.20–13.14; *P* < 0.001), and high AAC (HR, 4.32; 95% CI, 1.84–10.12; *P* < 0.001) **(**Table 4**)**.

**Table 4:** Risk factors for 5-year DSS

		Multivariate		
	N=516	HR	95% CI	P value
Age (>75)	123 (23.8%)	0.89	0.37-2.11	0.79
Sex (Male)	359 (69.6%)	1.02	0.39-2.68	0.971
pStage III	66 (12.8%)	6.26	2.94-13.36	<0.001
Histopathological type (Diffuse/mixed)	276 (53.5%)	1.38	0.65-2.93	0.407
Tumor location (Proximal)	136 (26.4%)	1.31	0.58-2.99	0.515
Surgical method （TG)	122 (23.6%)	5.38	2.20-13.14	<0.001
AAC (> 100)	214 (41.5%)	4.32	1.84-10.12	<0.001

*TG* total gastrectomy, *AAC* abdominal aortic calcification, *DSS* disease specific survival, *HR* hazard ratio, *CI* confidence interval

Univariate analysis identified high AAC, older age, male sex, high ASA-PS, low PNI, high CAR, pStage 3, proximal tumor, total gastrectomy, D2 dissection, postoperative complications, and adjuvant chemotherapy as predictors of poor RFS (Supplemental Table 3). Multivariate analysis revealed that older age (HR, 1.71; 95% CI, 1.02–2.84; *P* = 0.041), pStage 3 (HR, 2.84; 95% CI, 1.70–4.76; *P* < 0.001), total gastrectomy (HR, 2.76; 95% CI, 1.67–4.55; *P* < 0.001), and high AAC (HR, 2.63; 95% CI, 1.60–4.33; *P* < 0.001) were independent predictors of poor RFS **(**Table 5).

**Table 5:** Risk factors for 5-year RFS

		Multivariate		
	N=516	HR	95% CI	P value
Age (>75)	123 (23.8%)	1.71	1.02-2.84	0.041
Sex (Male)	359 (69.6%)	1.84	0.99-3.41	0.053
pStage III	66 (12.8%)	2.84	1.70-4.76	<0.001
Histopathological type (Diffuse/mixed)	276 (53.5%)	1.08	0.68-1.70	0.747
Tumor location (Proximal)	136 (26.4%)	1.13	0.68-1.87	0.644
Surgical method （TG)	122 (23.6%)	2.76	1.67-4.55	<0.001
AAC (>100)	214 (41.5%)	2.63	1.60-4.33	<0.001

*TG* total gastrectomy, *AAC* abdominal aortic calcification, *RFS* recurrence-free survival, *HR* hazard ratio, *CI* confidence interval

### Impact of AAC on the Site of Recurrence after Curative Resection of Gastric Cancer

**Fig. 3:**
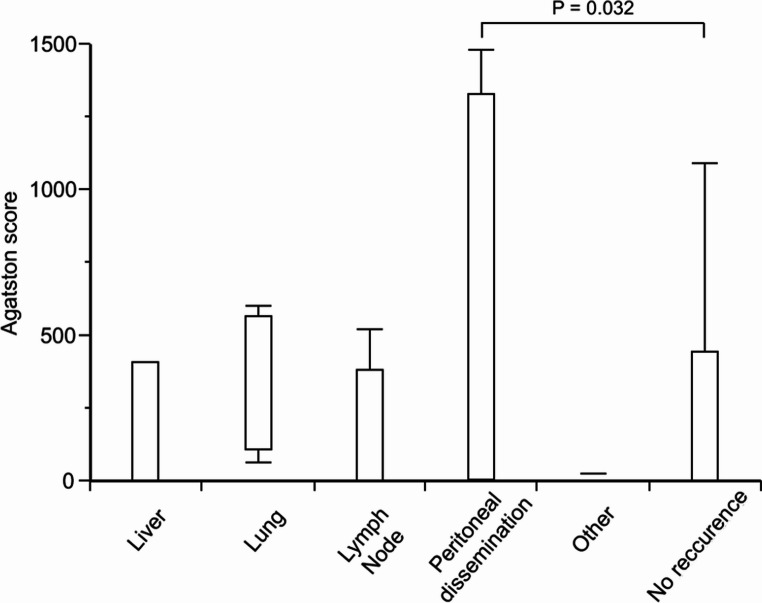
Distribution of Agatston scores according to initial site of recurrence

The association between AAC and the initial site of gastric cancer recurrence was evaluated. Among the 34 patients who experienced recurrence out of a total of 516 cases, peritoneal dissemination was the most frequent pattern (*n* = 13), followed by lymph node recurrence, liver metastasis, lung metastasis, and other sites (Table 6). No statistically significant difference was observed in the distribution of the initial recurrence sites between the low and high AAC groups (data not shown). Furthermore, stratified analysis based on the Agatston score revealed that patients with peritoneal dissemination had significantly higher Agatston scores than those without recurrence (*P* = 0.032, Fig. 3).

**Table 6:** Analysis of first recurrence site

	N = 34 / 516 (6.6%)
Liver	7 (1.4%)
Lung	4 (0.8%)
Lymph node	9 (1.7%)
Peritoneal dissemination	13 (2.5%)
Others	1 (0.2%)

## Discussion

This study introduced a practical method for predicting the postoperative prognosis of patients with gastric cancer by quantitatively assessing AAC volume preoperatively using CT scans.

Prior research has established a relationship between AAC and long-term outcomes in patients with liver cancer [21]. Furthermore, AAC has been linked to the long-term prognosis and recurrence of liver metastases in patients with stage II/III colorectal cancer [14]. Previous studies have identified an association between AAC and postoperative complications, such as suture failure, in patients with gastric cancer [13, 22]; however, to our knowledge, this study is the first to establish a relationship between AAC and the long-term prognosis of patients following gastric cancer surgery. While age, DM, and CAR are prognostic markers in patients with gastric cancer [23, 24], AAC may serve as a composite indicator of chronic inflammation and vascular aging, potentially reflecting the cumulative burden of these factors. Thus, AAC could offer additive prognostic value in preoperative assessment.

Vascular calcification arises from multiple mechanisms. For instance, inflammatory cytokines such as tumor necrosis factor-α promote the release of matrix vesicles from vascular smooth muscle cells [25]. These matrix vesicles accumulate calcium and phosphate ions, forming the core of hydroxyapatite crystal formation [26]. An important phenomenon related to the development of vascular calcification is the transformation of vascular smooth muscle cells (VSMCs) into osteoblast- or chondrocyte-like cells due to atherosclerosis [27]. During this transformation, VSMCs express transcription factors involved in osteogenesis, such as Runx2. Runx2 plays a key role in vascular calcification, and its absence in VSMCs suppresses their differentiation into bone or chondrogenic cells and inhibits calcification [28]. As such, aortic calcification is a consequence of chronic inflammation and atherosclerosis. In line with this notion, in the current cohort, patients with high AAC levels presented with underlying conditions such as DM, HT, and HL, were older and exhibited lower ASA-PS with elevated CAR levels.

Several studies have indicated that chronic inflammation and atherosclerosis are correlated with long-term prognosis following gastric cancer surgery [29]. Takemoto et al. demonstrated that CAR impacts short- and long-term outcomes in older patients with gastric cancer [30]. Additionally, prognostic factors such as the neutrophil-to-lymphocyte ratio [31] and modified Glasgow Prognostic Score [32] have been associated with the prognosis of patients with gastric cancer. In clinical practice, chronic inflammation and atherosclerosis affect the prognosis of gastric cancer. Szczepanik et al. demonstrated a correlation between interleukin (IL)−6 levels in the peripheral blood and the incidence of postoperative complications [33]. Nakata et al. reported that IL-2R levels influence postoperative outcomes [34]. Furthermore, Lin et al. observed elevated serum poly(A)-binding protein cytoplasmic 1 levels in patients with atherosclerosis-related transient ischemic attacks, and this marker increases the risk of mortality in patients with several cancer types [35]. Nevertheless, the molecular biological effects of chronic inflammation and atherosclerosis on gastric cancer remain unclear.

This study reports that aortic calcification might influence peritoneal dissemination recurrence after gastric cancer surgery. The peritoneal cavity contains many types of immune cells that can mediate direct contact with tumor cells exposed from the serous surface of the primary tumor. Although lymphocytes and macrophages are generally considered the major leukocytes of peritoneal immunity [36, 37], Kanemaru et al. found numerous neutrophil extracellular trap (NET)-like structures on the surface of human omental tissue removed by gastrectomy, indicating that NETs on the peritoneal surface may promote aggregation and proliferation of free tumor cells seeded in the abdomen [38]. Although it was not possible to directly link AAC to the tumor response of these immune cells at this time, arteriosclerosis and chronic inflammation often coexist in the background of patients with calcification, which may adversely affect immune responses to surgical invasion and the wound healing process, leading to the formation and persistence of NETs.

This study has some limitations that must be acknowledged. For instance, it was designed as a retrospective cohort study rather than a randomized controlled trial, which may affect the robustness of the findings. In the survival curves of this study, some early censored cases were observed. These were mainly due to early postoperative transfer to other hospitals. The number of cases was small, and their impact on the overall survival analysis results is thought to be limited. However, there are still limitations in terms of the completeness of follow-up. Additionally, conducting the analysis at a single institution may have limited the strength of the conclusions. Although the number of recurrence or death events was relatively small in the present analysis (*n* = 34), we applied an ROC analysis to exploratorily investigate a potential cut-off value for the Agatston score. Given the limited number of events, there is a possibility of overfitting, and further validation using an external dataset is warranted.

In addition, the Agatston score cutoff value used in this study is different from that used in similar studies on colorectal cancer [14]. It is said that the degree of aortic calcification varies depending on the height of the aorta [39], and the different cutoff values ​​may reflect this. The optimal cutoff value may be explored if similar studies are accumulated.

In conclusion, this study supports a strong association between AAC and long-term postoperative prognosis in patients with gastric cancer. Future multicenter prospective studies with larger cohorts of patients exhibiting high AAC levels are essential to examine the impact of AAC on postoperative outcomes.

Our findings suggest that preoperative evaluation of abdominal aortic calcification using the Agatston score may serve as a useful, non-invasive imaging biomarker to stratify patients by risk. This could help inform postoperative surveillance strategies and shared decision-making in the clinical management of gastric cancer.

## Supplementary Information

Supplementary Material 1 (XLSL 10.7 KB)

Supplementary Material 2 (XLSL 10.8 KB)

Supplementary Material 3 (XLSL 10.7 KB)

## Data Availability

The datasets generated during and/or analyzed during the current study are available from the corresponding author on reasonable request.
